# Children and adolescents with speech sound disorders are more likely to have orofacial dysfunction and malocclusion

**DOI:** 10.1002/cre2.602

**Published:** 2022-06-20

**Authors:** Åsa Mogren, Anders Sand, Christina Havner, Lotta Sjögreen, Anna Westerlund, Monica Barr Agholme, Anita Mcallister

**Affiliations:** ^1^ Department of Clinical Science, Intervention and Technology (CLINTEC), Division of Speech and Language Pathology Karolinska Institutet Stockholm Sweden; ^2^ Public Dental Service Mun‐H‐Center, Orofacial Resource Centre for Rare Diseases Gothenburg Sweden; ^3^ Department of Orthodontics, Institute of Odontology, Sahlgrenska Academy Gothenburg University Gothenburg Sweden; ^4^ Department of Dental Medicine, Division of Orthodontics and Pediatric Dentistry Karolinska Institutet Stockholm Sweden; ^5^ Medical Unit Speech and Language Pathology, Women's Health and Allied Health Professionals Theme Karolinska University Hospital Stockholm Sweden

**Keywords:** bite force, chewing efficiency, intraoral sensory‐motor function, Nordic Orofacial Test‐Screening

## Abstract

**Background:**

Children with speech sound disorders (SSD) form a heterogeneous group that differs in terms of underlying cause and severity of speech difficulties. Orofacial dysfunction and malocclusions have been reported in children with SSD. However, the association is not fully explored.

**Objectives:**

Our aims were to describe differences in orofacial function and malocclusion between a group of children and adolescents with compared to without SSD and to explore associations between those parameters among the group with SSD.

**Methods:**

A total of 105 participants were included, 61 children with SSD (6.0–16.7 years, mean age 8.5 ± 2.8, 14 girls and 47 boys) and 44 children with typical speech development (TSD) (6.0–12.2 years, mean age 8.8 ± 1.6, 19 girls and 25 boys). Assessments of orofacial function included an orofacial screening test and assessment of bite force, jaw stability, chewing efficiency, and intraoral sensory‐motor function. Possible malocclusions were also assessed.

**Result:**

Children with SSD had both poorer orofacial function and a greater prevalence of malocclusion than children with TSD. Furthermore, children with SSD and poorer orofacial function had a greater risk of malocclusion.

**Conclusion:**

Our result suggests that children with SSD are more prone to having poorer orofacial function and malocclusion than children with TSD. This illustrates the importance of assessing coexisting orofacial characteristics in children with SSD, especially since orofacial dysfunction may be linked to an increased risk of malocclusion. This result highlights the need for a multiprofessional approach.

## 1 BACKGROUND

### 1.1 Speech sound disorders

Speech sound disorders (SSD) are one of the most common neurodevelopmental disorders, with a reported occurrence between 2% and 13% in children aged 6–8 years (Shriberg, 2010; Shriberg et al., 1999). The umbrella term SSD includes difficulties with articulation, phonology, and motor speech disorders (Waring & Knight, 2013). An SSD is regarded as persistent when the speech difficulties persist at ages when all speech sounds are mastered in typically developing children. In Swedish, all consonants, including/r/and/s/sounds, are established by the age of 6 years (Blumenthal & Lundeborg Hammarström, 2014).

Children with SSD form a heterogeneous group that differs in terms of underlying cause and severity (Waring & Knight, 2013). Previous research has suggested that speech difficulties rarely exist in isolation (Gillberg, 2010), but rather that general motor difficulties are common in children with SSD of varying etiology (Green & Nip, 2010; Hill, 2010; Mogren et al., 2020; Visscher et al., 2007). The growing body of research on the genetic causes of SSD also points to a shared foundation for several neurodevelopmental disorders (Eising et al., 2018).

### 1.2 Orofacial function

Typically developing children have acquired good oral motor control before the age of 4 (Martinez & Puelles, 2011), but development continues and is further refined throughout childhood, especially for speech. Deviant or delayed general development often includes orofacial dysfunction (Bergendal et al., 2014) and this is a common symptom in syndromes and rare diseases (Sjogreen, Mogren, et al., 2015). Orofacial dysfunction may affect many functions of importance for the quality of life, in addition to speech, for example, facial expression, voice, resonance, feeding, chewing and swallowing, saliva control, intra‐, and extraoral sensory function, and nose breathing (Sjogreen, Mogren, et al., 2015).

Although several studies have reported coexisting gross and fine motor skills among children with SSD (Green & Nip, 2010; Hill, 2010; Visscher et al., 2007), only a few have specifically described orofacial function in children with SSD. Some studies have reported that children with SSD may exhibit reduced stability and control of the jaw (Mogren et al., 2021; Namasivayam et al., 2013; Terband et al., 2013) which is a prerequisite for efficient articulatory positions in the mouth and for lip and tongue movements involved in speech production (Wilson & Nip, 2011). Thus, there is some evidence that children with SSD may have coexisting orofacial dysfunction, but the research so far has been sparse. One reason could be the historical lack of proper methods to investigate orofacial function (Green & Nip, 2010). However, now several different assessment protocols and instrumental methods for objective evaluation of orofacial function exist.

### 1.3 Malocclusion

Occlusal development is affected by a number of genetic and environmental factors (Linder‐Aronson, 1970; Ovsenik et al., 2007). Sjögreen, Andersson‐Norinder, et al. (2015) reported a higher prevalence of malocclusions in individuals with rare diseases and orofacial dysfunction than in individuals with rare diseases but without orofacial dysfunction. Furthermore, some oral habits and tongue protrusion are more common among individuals with open bites and posterior crossbites (Dimberg et al., 2013).

Malocclusion can affect different orofacial functions: Good occlusal contacts are important to prepare the bolus (Fontijn‐Tekamp et al., 2000) and provide good chewing efficiency (Magalhães et al., 2010). An open bite may result in interdental production of dental fricatives and the articulation of labiodental fricatives may be affected by prenormal occlusion (Profitt, 2013). Oppositely, orofacial dysfunction may also influence occlusal development (Behlfelt et al., 1990; Linder‐Aronson, 1970). Foletti et al. (2018) also saw an association between atypical swallowing patterns and relapse after orthognathic surgery. Several studies have shown that a reduced oral muscular strength may negatively influence facial growth (Kiliaridis et al., 1995; Kiliaridis & Katsaros, 1998) and that a posterior crossbite may develop due to a low position of the tongue (Ovsenik, 2009).

Malocclusions are common in individuals with neurodevelopmental disorders (de Castilho et al., 2018; Fontaine‐Sylvestre et al., 2017). However, few studies have investigated whether children with SSD may be more prone to having malocclusions. Our first hypothesis was that children with SSD would, as a group, have a poorer orofacial function and would have malocclusions more frequently than their typical speech development (TSD) peers. Since orofacial function and malocclusions are connected (Kiliaridis et al., 1995; Kiliaridis & Katsaros 1998; Ovsenik, 2009), our second hypothesis was that among the group with SSD, individuals with poorer orofacial function would be more at risk of having malocclusions.

### 1.4 Aims

We aimed to describe differences in orofacial function and the occurrence of malocclusion between groups of children and adolescents with and without SSD. We further aimed to explore associations between orofacial function and malocclusion in this patient group.

### 1.5 Research questions

- How do children and adolescents with SSD differ from those with TSD regarding orofacial function, including bite force, jaw stability, chewing efficiency, and intraoral sensory function?
- Is there a relationship between orofacial dysfunction and malocclusion in children and adolescents with SSD?

## 2 METHODS

This is a prospective cross‐sectional design study performed in a clinical setting at an orofacial resource center with referred patients with SSD and a control group of participants with TSD.

### 2.1 Participants

Participants with SSD were referred for a speech and oral motor examination between 2014 and 2016. All consecutive patients who met the inclusion criteria were offered participation in the study. The inclusion criteria were SSD persisting after the age of 6 years, no moderate or severe intellectual disability, cerebral palsy, and/or severe autism spectrum disorder. All but one teenager accepted to participate. The total number of participants with SSD was 61 children and adolescents (hereafter referred to only as “children” for convenience) (Table 1). The participants with SSD had varying degrees of speech difficulties. They were all clinically assessed by a speech‐language pathologist as having motor speech involvement and had impaired consonant production with no single consonant established in all children (see Supporting Information: Table 1 for further information on speech characteristics and differential diagnostic procedure). Five participants were raised in bilingual homes but had Swedish as their first language and two children were adopted internationally at 2:6 and 3 years of age. Three sibling pairs were included. All participants but one followed the regular curriculum for compulsory schooling.

**Table 1:** Background information, orofacial function, and malocclusion in children with speech sound disorders (SSD) and children with typical speech development (TSD)

Variable	Children with SSD (*n* = 61)	Children with TSD (*n* = 44)
*Background information*		
Age, year:month, mean ± SD	8:5 ± 2:8	8:7 ± 1:6
Sex: Females/males	14/47	19/25
Percent consonants correct,^a^ mean ± SD	66 ± 22	100 ± 0
*Orofacial function*		
NOT‐S^b^ total score (0–12), mean ± SD	4.0 ± 2.2	0.25 ± 0.49
Maximum bite force, Newton, mean ± SD	235 ± 123	346 ± 116
Jaw stability,^b^ bite block level (0–6) mean ± SD	3.4 ± 1.3	5.3 ± 0.87
Chewing efficiency,^c^ SD Hue, mean ± SD	0.20 ± 0.11	0.14 ± 0.06
Sensory‐motor function,^b ^(0–8), mean ± SD	5.8 ± 1.9	7.05 ± 0.94
*Malocclusion*		
No malocclusion *n* (%)	24 (39)	31 (70)
Malocclusion^d ^ *n* (%)	37 (61)	13 (30)
Class II (postnormal) *n* (%)	15 (25)	8 (18)
Class III (prenormal) *n* (%)	9 (15)	0
Deep bite *n* (%)	14 (23)	2 (4)
Posterior crossbite *n* (%)	13 (21)	3 (7)
Anterior open bite *n* (%)	7 (12)	0

Abbreviations: NOT‐S, Nordic Orofacial Test‐Screening; SD, standard deviation; SD Hue, the standard deviation of the variance of Hue.

^a^ Speech production was assessed using the Swedish Articulation and Nasality Test (Lohmander et al., 2017). Consonants were scored as correct or incorrect, according to instructions in Shriberg et al. (1997). The speech production results are described in Mogren et al. (2020).

^b^ Ordinal scale data, for a more detailed distribution of data, see Figure 1.

^c^ One missing value in each group.

^d^ Some occlusal traits co‐occur in some children therefore the number of malocclusions does not match the number of children assessed as having a malocclusion.

The inclusion criteria for the participants with TSD were TSD, no known neurodevelopmental disorder, and age between 6 and 18 years. They were recruited from the Public Dental Health Service. A total of 44 children with TSD participated (Table 1).

### 2.2 Procedure and test items

All assessments were performed by a speech‐language pathologist and orthodontist in a clinical setting. The dental examination was performed in a dental chair and the other assessments were performed with the participant seated in an ergonomic work chair while being videotaped (Canon Legria HF S11; Canon, Japan, with an external microphone, Canon DM‐100; Canon). The camera was placed 1.5–2 m from the child on the opposite wall.

### 2.3 Assessments of orofacial function

#### 2.3.1 Screening of orofacial function

The general orofacial function was assessed with the nordic orofacial test‐screening (NOT‐S) (Bakke et al., 2007). The NOT‐S is a validated screening test and is regarded as a comprehensive test that covers several orofacial functions. It consists of a structured interview and a clinical examination. The screening is divided into 12 domains: sensory function, breathing, habits, chewing and swallowing, drooling, dryness of the mouth is assessed in a structured interview and face at rest, nose breathing, facial expression, masticatory muscles and jaw function, oral motor function, and speech in the clinical examination. One or more positive answers in a domain generate a “dysfunction score.” The maximum NOT‐S score is 12. Typically developing children (>5 years) have a mean score of <2 (McAllister & Lundeborg, 2013).

#### 2.3.2 Bite force

The maximum voluntary bite force could be an indicator of the functional state of the masticatory system (Koc et al., 2010). Measurements of bite force were performed with an occlusal force meter (Occlusal Force‐Metre GM 10; Nagano Keiki Co., Japan). The biting element was placed on the first molar and the participant was instructed to bite down as hard as possible. The measurement was repeated three times on each side. To calculate a mean value for each participant, the greatest value on each side was added up and then divided by two to generate a mean value. The biting element that is used in the occlusal force meter is developed so that children at age 6 or older are expected to be able to carry out the assessment. Owais (et al. 2013) found a mean maximum bite force of 433 N in 10‐year‐old typically developing children in the late mixed dentition stage.

#### 2.3.3 Jaw stability

Jaw grading bite blocks (Rosenfeld‐Johnson, 2005) were used to assess jaw stability. Six bite blocks of different sizes were used in a stepwise manner. The assessment started with the smallest bite block (No. 2). Participants were asked to hold the bite block between the molar teeth for 15 s on each side. The examiner attached the bite block to a gauge meter and pulled the bite block gently, carefully monitoring the pull to be exactly 1 kN on the gauge meter while measuring and counting the seconds out loud. The assessment was finished when the participant was unable to retain the bite block. If the participant managed to hold the bite block for 15 s, the examination was moved on to the next size of the bite block. The largest bite block that the participant was able to retain formed their jaw grading result. The minimum value on this task was size 2 and the maximum was 6.

#### 2.3.4 Chewing efficiency

To assess chewing efficiency, a two‐colored chewing gum test was used (Halazonetis et al., 2013; Schimmel et al., 2007). By using digital image processing software (Viewgum), the analysis quantifies how well the colors have been mixed, resulting in a measure of chewing efficiency. A two‐colored chewing gum specifically developed for this purpose was used (Firmenich, Switzerland). The participants were asked to chew 30 times on the chewing gum and then spit it out in a cup. Chewing strikes were counted out loud by the examiner. Each side of the flattened gum was scanned in a flatbed scanner (resolution 600 dpi) and analyzed with the Viewgum software (ViewGum©software; dHAL Software, Greece, www.dhal.com). Chewing efficiency was measured as the standard deviation of the variance of Hue (SD Hue), which is a measure of how well the two colors have been mixed. A low SD Hue value indicates better masticatory performance. In a study by Kaya et al. (2017), 25 healthy children with mixed dentition and a mean age of 9:7 years had an SD Hue mean value of 0.512 after chewing 20 strokes on a two‐colored chewing gum. The 27 included adults in the same study had an SD Hue mean value of 0.382. In a study by Halazonetis et al. (2013), healthy adults without malocclusion had an SD Hue value of 0.05 after chewing 30 strokes. Another chewing gum was used in both these studies, which means that the values are not completely comparable to the values in this study.

#### 2.3.5 Intraoral sensory‐motor function

When testing intraoral stereognosis – a measurement of intraoral sensory‐motor function, figures of different shapes and sizes are used. However, no standardized procedure is available (Boliek et al., 2007). Oral stereognosis tests the ability to recognize different shapes in the mouth. In this study, a set of four different shapes in two different sizes was used (star, triangle, circle, half‐circle) (Byteme AB, Gothenburg, Sweden). The assessment was performed as described in STockholm ORalMotoriska bedömningsprotokoll (Henningsson et al., 2007). The stainless‐steel figures were attached to a nylon thread. Participants were asked to open their mouths and close their eyes. The figure was placed on the tongue by the examiner. The participant was then asked to close the mouth and “feel the shape of the figure.” The figure was removed after 10 s, and the participant was asked to point to pictures of the figures to determine which picture matched the figure in the mouth. The maximum score was 8 when all the figures were correctly identified. Typically developing children between the age of 6–8 years is expected to perform very well on this assessment, already at the age of 6, a “ceiling effect” was described (Andersson & Buhr, 2009 (in Swedish)).

### 2.4 Assessment of malocclusion

Intra‐ and extraoral examinations together with occlusal records were taken by an orthodontist. Clinical photographs were taken for reliability testing. Due to the comprehensive number of tests and examinations, no intra‐ or extraoral radiographs were taken, nor dental casts. The incidence of malocclusion was based on the objective orthodontic treatment need. For this, the index of orthodontic treatment need, dental health component (IOTN‐DHC) (Brook & Shaw, 1989), was used, which is a well‐known and widely used index for patient selection in orthodontic clinics. The IOTN‐DHC uses a 5‐point scale where the cutoff points have been defined precisely. The patient is assigned to a group based on severity according to the index. One means no treatment need, 3 means borderline, and 5, is a severe treatment need. Groups 1 and 2 together with 3d (space anomalies) was assessed as not having a malocclusion whereas 3abcef, 4, and 5 were recorded as having malocclusion.

### 2.5 Reliability

Inter‐ and intrarater reliability testing was performed for the NOT‐S and malocclusions assessments. The assessors were blinded to the participant group. Reliability for the other assessments could not be tested, as they were performed in a clinical setting and dependent on live assessment.

#### 2.5.1 Malocclusion

Inter‐ and intrarater agreement on the presence of malocclusion was calculated by Kappa statistics. Reassessment of the interrater agreement was performed for all participants. Intrarater agreement was based on the reassessments of 20% of the participants through random selection. Both interrater agreement (*κ* = 0.844) and intrarater agreement (*κ* = 0.901) was estimated as very good.

#### 2.5.2 Nordic Orofacial Test‐Screening

Recordings of 31% of the NOT‐S assessments were randomly selected and reassessed. Three items could not be assessed from video recordings alone and were, therefore, excluded from the reliability assessment (nose breathing, palpation of the jaw muscles, and intraoral examination of the soft palate). Inter‐ and intrarater agreement varied between good and excellent. The interrater agreement had a median of 90% (79%–100%) point‐by‐point agreement and the intrarater agreement had a median of 95% (90%–100%) point‐by‐point agreement.

### 2.6 Statistical analysis

Descriptive statistics were used to characterize the orofacial function and dental characteristics among the children with SSD and TSD. Where relevant, groups were compared using relative risks (RR), which describe how much greater the risk of a particular outcome is in one group compared to another. Separate logistic regression analyses were used to describe the relationship between orofacial function and malocclusion among the children with SSD. The result of this analysis is presented both in the odds‐ratio metric used in the logistic model, and also back‐transformed to the probability scale so that it will be easier to see the risk of malocclusion associated with a particular degree of orofacial function. This analysis focused only on the children with SSD, as most children with TSD had a typical orofacial function and no malocclusion. Including children with TSD in this analysis would thus erroneously inflate the strength of any relationship. We focused the inferential statistics on confidence intervals (CIs) rather than statistical significance testing (Wasserstein et al., 2019). A CI should be interpreted as a “compatibility interval” illustrating what parameter values are most compatible with the data (Amrhein et al., 2019). All analyses were carried out in R (R Core Team).

## 3 RESULTS

### 3.1 How do children with SSD and children with TSD differ regarding orofacial function?

**Figure 1:**
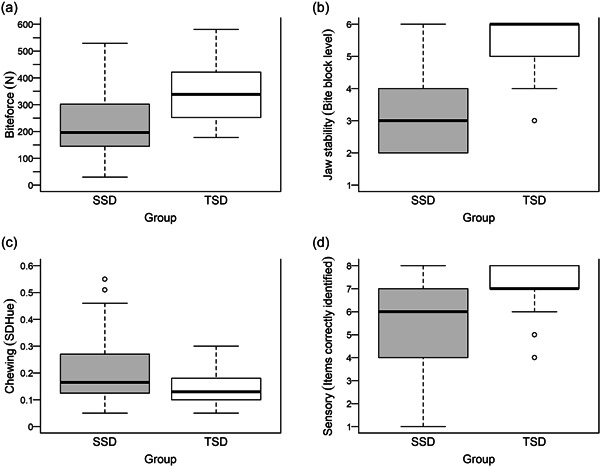
Boxplots describing orofacial function (Biteforce (a), Jaw stability (b), Chewing efficiency (c), Sensory function (d)) among the children with SSD (gray) and children with TSD (white). Boxplots illustrate quantiles with the thick black line illustrating the median, the upper and lower limit of the box illustrate the 25th and the 75th percentile, and the lower and upper whisker plots indicate the minimum and maximum values, except “extreme values,” which are marked as separate circles. If the boxes and whiskers are roughly symmetrical around the median, the sample is roughly normally distributed on that variable, as is mostly the case for the SSD participants. SD Hue, the standard deviation of the variance of Hue; SSD, speech sound disorders; TSD, typical speech development.

Table 1 and Figure 1 present information on all assessments of orofacial function for both groups. On the basis of NOT‐S, children with SSD generally displayed orofacial dysfunctions in several domains, whereas children with TSD did not. In line with the study by McAllister and Lundeborg (2013), only one child with TSD had a total NOT‐S score of 2 and all other TSD children had total NOT‐S scores of 0 or 1. In contrast, 53 children with SSD (87%) had total NOT‐S scores of ≥2. These results are more thoroughly described in Mogren et al. (2020).

When assessing jaw stability, many children with TSD achieved the highest level (bite block no. 6) or the second‐highest bite block level (bite block no. 5) (54% and 31%, respectively). In comparison, only a few children with SSD reached those levels (10% and 11%, respectively). On the intraoral stereognosis test, 77% of the children with TSD correctly identified eight or seven out of eight figures (36% and 41%, respectively). In comparison, 41% of children with SSD correctly identified eight or seven figures (21% and 20%, respectively).

The only differences in performance related to age and sex in the TSD group were found in bite force, where girls and younger children had somewhat lower values (see Supporting Information: Table 2).

### 3.2 How do children with SSD and children with TSD differ regarding malocclusions?

Two‐thirds of the children with SSD had malocclusion (*n* = 37; 61%) (Table 1) compared to 29% (*n* = 13) of the children with TSD. Thus, there was a 2.22 times greater risk of malocclusion for the children with SSD compared to children with TSD, RR (95% CI [1.32, 3.75]). Also, class III relation and anterior open bite were detected only in the SSD group (Table 1).

### 3.3 Is there a relationship between orofacial function and malocclusions in children with SSD?

The relationship between orofacial function and malocclusion was described using separate logistic regression analysis. The different assessments of orofacial function were correlated. The three strongest correlations were between the high NOT‐S total score and weaker bite force (Pearson product‐moment correlation), *r* = −.52, and poorer sensory function, *r* = −.47. Individuals with weaker bite force also had poorer jaw stability, *r* = .65. Thus, it is not possible to provide an exact estimate of the impact of any individual assessment of dysfunction (as each estimate will also include the impact of the other assessments).

**Figure 2:**
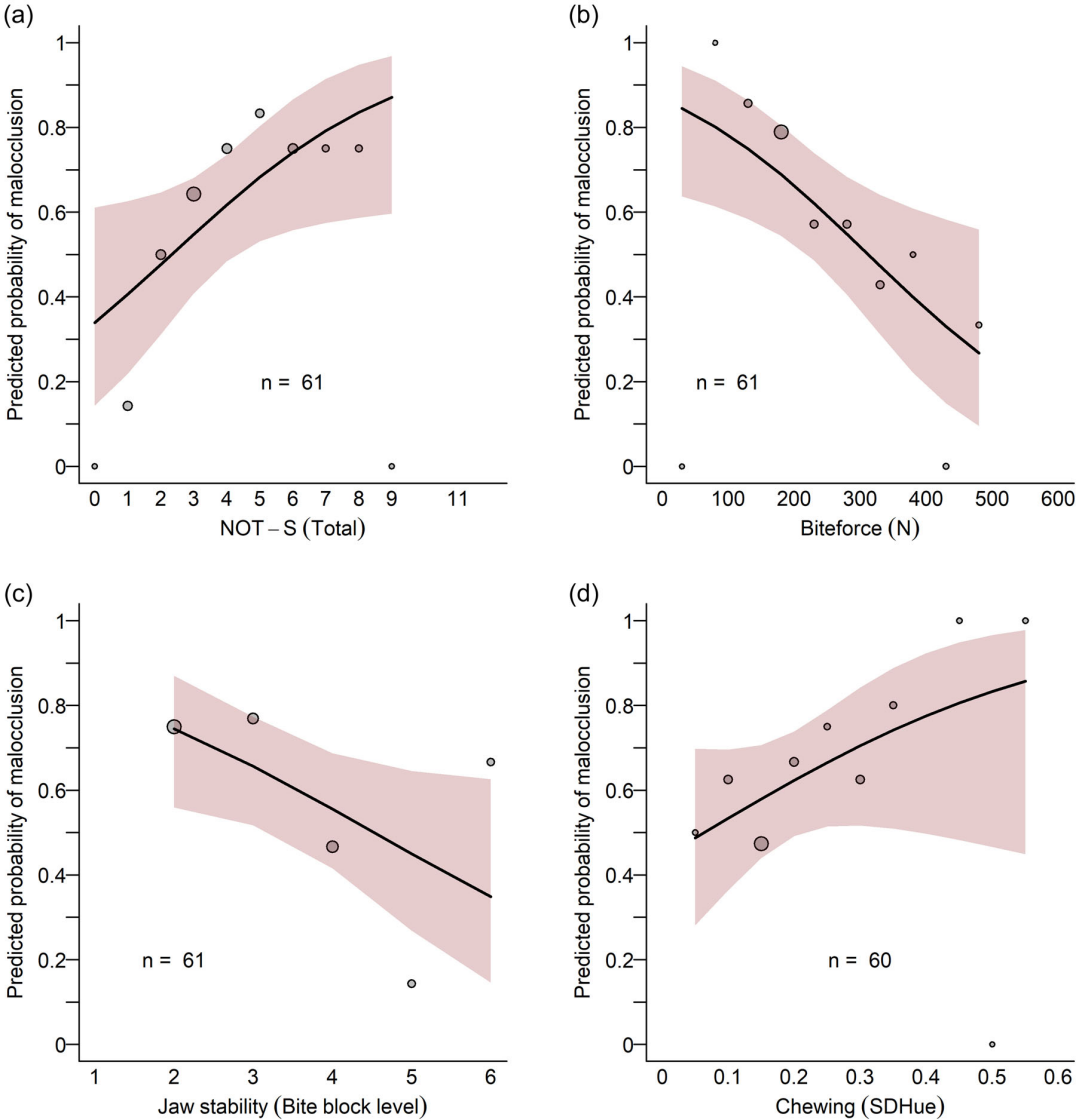
Illustration of the logistic regressions between malocclusion and NOT‐S (a), bite force (b), jaw stability (c), and chewing efficiency (d) in children with SSD. In each panel, the logistic regression function has been back‐transformed to the probability scale (*y*‐axis). The solid black curve illustrates the *output of the logistic regression model* describing the probability of a malocclusion (*y*‐axis) for a particular predictor value *(x*‐axis). The light red area illustrates the 95% CI around the regression function. We also want to communicate *the empirical data*, that is, the empirical proportion of malocclusion for different children along with the predictor variable (*x*‐axis). As the predictors are continuous variables, we have to simplify the communication and bin the participants into different groups along the *x*‐axis (e.g., 100–150 N). Each bin is illustrated by a circle in the figures. The circles' position on the *x*‐axis is the group's binned predictor value and its position on the *y*‐axis is the actual proportion of participants in that group that had a malocclusion. The size of the circle is proportional to how many participants were included in that group. Note that the logistic regression model was built upon the continuous data of the predictor variables, the binning is only for illustrative purposes in this figure. Our aim is to communicate that the regression function aligns with the empirical data. CI, confidence interval; NOT‐S, Nordic Orofacial Test‐Screening; SSD, speech sound disorders.

Table 2 describes the logistic regression models with each separate orofacial function as a predictor. The strongest relationship between orofacial function and malocclusion was for the NOT‐S total score. Figure 2a illustrates this relationship. In this figure, the logistic regression output has been back‐transformed to the probability scale (*y*‐axis). The black curve describes the logistic regression function specifying the probability of having a malocclusion (*y*‐axis) for an individual with a particular NOT‐S score (*x*‐axis). At a NOT‐S total score of 2, the model predicts a 48% risk of having a malocclusion, whereas, at a NOT‐S score of 6, the model predicts a 74% risk of having a malocclusion. For NOT‐S, the odds ratio of having a malocclusion increased by 1.33 (95% CI [1.03, 1.78]) for each additional NOT‐S score. Similarly, as shown by the relatively steep slope in Figure 2, bite force and jaw stability were predictive of the risk of having a malocclusion. In comparison, chewing efficiency was less related to the risk of malocclusion (illustrated by the flatter slope in the relationship between chewing and probability of a malocclusion), as was intraoral sensory‐motor function (not illustrated in Figure 2, but the coefficients are reported in Table 2). Due to the moderate interindividual difference in age in the sample, we also examined these relationships when controlling for age. However, the regression results remained virtually unchanged for all orofacial functions, see Table 2.

**Table 2:** Coefficients and 95% CI, in square brackets, for each of the separate logistic regression models that predicted malocclusion based on different orofacial function variables, unadjusted and adjusted for age, respectively

Orofacial function variable	Unadjusted model		Adjusted for age	
	Intercept	Coefficient	Intercept	Coefficient
NOT‐S (*per* total score)	0.51	1.33 [1.03, 1.78]	0.96	1.31 [1.01, 1.75]
Biteforce (*per* 100 N)	6.53	0.55 [0.33, 0.86]	5.96	0.54 [0.31, 0.89]
Jaw stability (*per* bite block level)	6.82	0.65 [0.42, 0.98]	7.55	0.67 [0.41, 1.04]
Chewing (*per* 0.1 SD Hue)	0.79	1.45 [0.88, 2.59]	1.89	1.34 [0.80, 2.46]
Intraoral sensory‐motor function (*per* item correctly identified)	2.79	0.90 [0.67, 1.19]	4.81	0.94 [0.69, 1.26]

*Note*: In this table, we report the coefficients in the odds ratiometric (the exponents of the logit‐coefficients). See Figure 2, for a graphical presentation of the unadjusted regression models when the output has been transformed to the probability scale.

Abbreviations: CI, confidence interval; NOT‐S, Nordic Orofacial Test‐Screening; SD Hue, the standard deviation of the variance of Hue.

**Figure 3:**
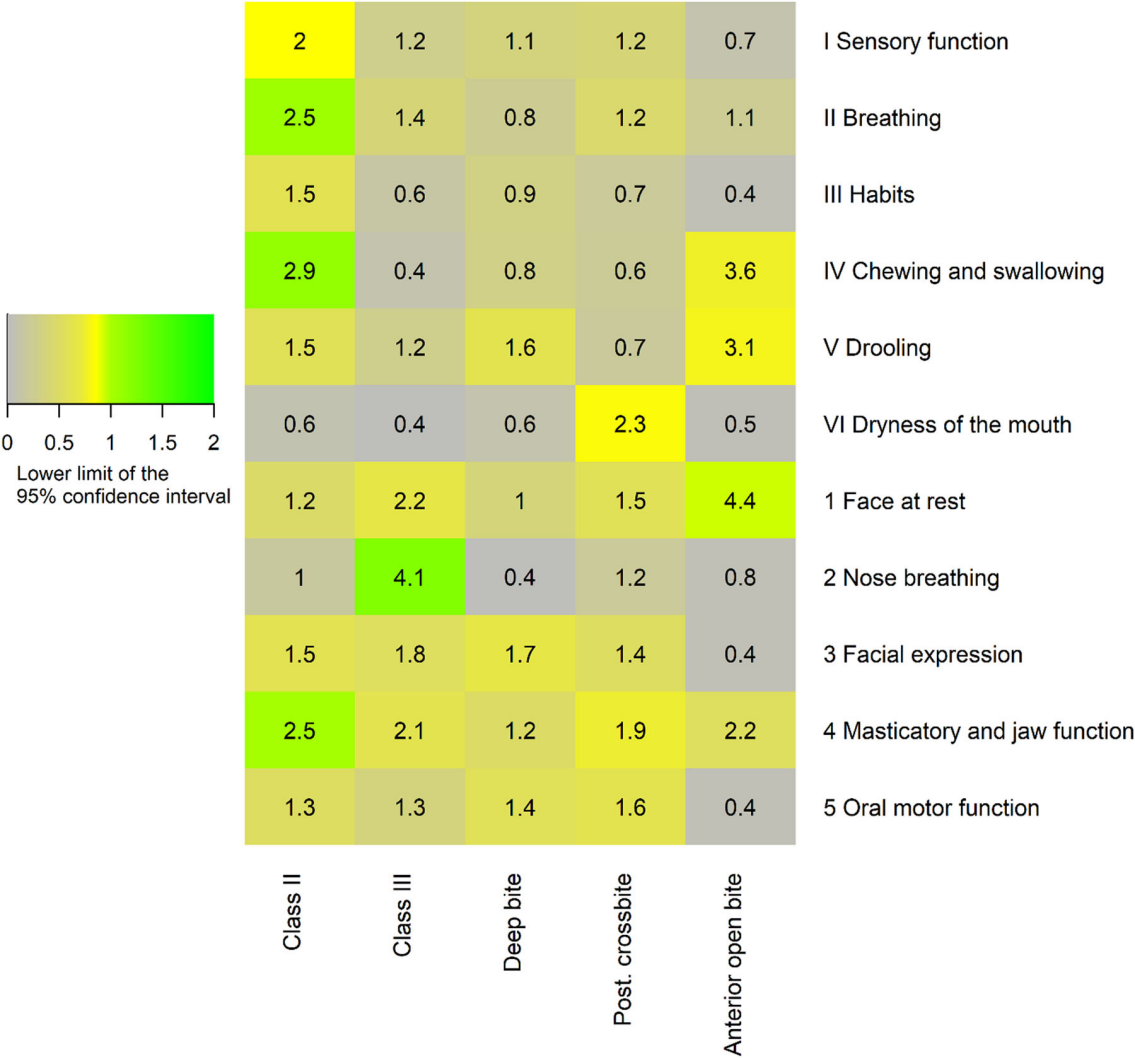
A “heat map” listing the relative risk of having the malocclusion listed in the column based on having a positive answer on the NOT‐S domains listed in the rows. The number is the relative risk. The color scale is based on the lower limit of a 95% CI around the relative risk; in this way, the coloring indicates the confidence of the relationship is strong, rather than the strength of the relationship. See the main text for more information. CI, confidence interval; NOT‐S, Nordic Orofacial Test‐Screening.

To gain a more detailed description of the relationship between each NOT‐S domain and the risk of having a particular type of malocclusion, we calculated the RR of having a particular type of malocclusion depending on whether the NOT‐S domain had a dysfunction score or not (a positive response). The result of this exploratory inquiry is presented in Figure 3. For example, there was an increased risk of having an open bite for individuals with a positive “Face at rest” response compared to individuals with a negative response. Five of the 22 individuals with a positive response had an open bite (23%), whereas 2 out of the 39 individuals with a negative response in this domain had an open bite (5%), a 4.43 times greater risk (95% CI [0.94, 20.97]). Other noteworthy relationships were between “Nose breathing” and Class III and between “breathing,” “chewing and swallowing,” and “masticatory and jaw function,” and having a class II malocclusion.

## 4 DISCUSSION

### 4.1 Differences between children with SSD and TSD regarding the orofacial function

The children in the SSD group were mainly referred to the clinic for assessment of speech difficulties and had no known other neuromuscular or neurological diseases. Despite this, the participants with SSD generally had poorer orofacial function than the participants with TSD. In the NOT‐S assessment, “Masticatory and jaw function” and “Chewing and swallowing” were the most affected domains in children with SSD. As jaw instability may lead to difficulties with the differentiated lip and tongue movements required for speech (Wilson & Nip, 2011), poorer orofacial functioning may be linked to difficulties with speech development. A third of the children with SSD (31%) had difficulties with the assessment of intraoral sensory‐motor function (oral stereognosis) compared to only two children (4%) in the TSD group. Intraoral sensory‐motor function is also regarded as important for both speech sound development (Crary et al., 1981) and chewing (Peyron et al., 2002) which again suggests a link between orofacial function and the children's speech, feeding, and eating development.

Our result is in line with a sparse but growing body of literature suggesting that orofacial dysfunction is more prevalent in children with SSD/motor speech disorders (Grigos & Kolenda, 2010; Mogren et al., 2021; Namasivayam et al., 2013; Terband et al., 2013). Potter et al. (2019), for example, reported reduced tongue strength in children with motor speech disorders compared with children with TSD developmental speech sound errors. Similar findings come from studies with different methodological approaches. Wren et al. (2016) and Stein et al. (2020) longitudinally studies children with SSD and found that week sucking and similar early fine motor coordination problems were important predictors of persistent and severe SSD.

### 4.2 Relationship between orofacial function and malocclusions in children with SSD

In our sample of children with SSD, those with poorer orofacial function were more likely to have a malocclusion. These results are in line with Sjogreen et al. (2010), who reported a higher prevalence of malocclusions in individuals with rare diseases and orofacial dysfunction and that individuals with neuromuscular disorders with reduced muscular strength and hypotonia had a higher prevalence of open bite malocclusions. Our results also agree with earlier studies by Kiliaridis et al. (1995, 1998) who showed that reduced oral muscular strength can influence facial growth.

A more detailed analysis of NOT‐S items revealed that low activity in the mm masseter during biting and an open mouth at rest were both associated with an increased risk of having malocclusion, a class III malocclusion, and, most of all, open bite. D'Onofrio (2019) has suggested that mouth breathing encourages a low jaw position, but it is possible that it could also work the other way around, that a low jaw position encourages mouth breathing because of the open mouth position. If the jaw is held in a low position, a low lingual resting position is also likely and this tongue position has previously been correlated with class II and class III relationships (Souki et al., 2009). The association between the dysfunction score on the domain of “Breathing” (snoring) and a class II malocclusion is in line with earlier research (Pirilä‐Parkkinen et al., 2009).

## 5 METHODOLOGICAL CONSIDERATIONS

Difficulties in oral motor performance have been reported in children with SSD (Hill, 2001; Mogren et al., 2020; Visscher et al., 2007) but rarely described in detail. This may be because methods to assess orofacial function are often based on observations. Braden et al. (2019) found that despite bilateral perisylvian polymicrogyria being a well‐known cause of severe orofacial dysfunction, not 1 of the 48 studies they reviewed used formal assessment tools to accurately measure oral structural or functional deficits. In this study, a variety of methods were used to gain a comprehensive and objective description of the orofacial function. For example, both bite force and chewing efficiency were measured in novel quantitative ways. We believe our study reveals the importance of performing assessments of orofacial function with objective and reliable tests and instruments.

As the NOT‐S assesses both sensory and motor function and is a combination of background information (interview) and examination findings we believe that this test is a sensitive measure of orofacial function. This is supported by our findings that many children with SSD display orofacial dysfunctions on the NOT‐S test. Although several earlier studies have assessed intraoral sensory function, there is no consensus about which method is the most reliable and valid (Boliek et al., 2007). Clinical assessments of intraoral sensory function commonly use 2‐point discrimination tasks and/or oral stereognosis (Jacobs et al., 2002). In this study, sensory‐motor function was assessed both through an anamnestic questionnaire (the NOT‐S interview) and a clinical assessment (OS), these two measures assess different aspects of sensory function and thus increase validity.

## 6 LIMITATIONS

The children in the SSD group were all referred to the orofacial resource center for a speech and oral motor examination which may have influenced the results. In other words, the exact degree of orofacial dysfunction or frequency of malocclusion found in our group of children with SSD may not be fully representative of the patient group at large.

In comparing the group of children with SSD to the group of children with TSD, we need to note that they were similar in age but not exactly similar in the sex distribution. This is in line with the reported prevalence of SSD among boys and girls (Dockrell et al., 2014; Wren et al., 2016), there was a slightly higher ratio of boys among our participants with SSD as compared to the participants with TSD. However, in previous studies of orofacial function, no differences between boys and girls have been found. Noterdaeme et al. (Noterdaeme et al., 2002) found no sex differences in motor performance in children with language disorders or in children with high functioning autism and Sjogreen et al. (2010) found no sex differences in orofacial function in individuals with rare diseases. As such, we do not believe that the difference in sex ratio between the two groups substantially influenced our results.

In analyzing the relationship between orofacial function and malocclusion, all measures of orofacial function were correlated and the impact of any one measure alone could not be estimated. In practice, this statistical issue is probably not important, as few children had, for instance, a very poor bite force but normal jaw stability. Furthermore, we framed our statistical analysis so that orofacial function was used to statistically predict the risk of malocclusion (e.g., was there a greater risk of malocclusion for individuals with a weak bite force?) and we have framed our discussion of the results accordingly. We should, of course, clarify that assessments of orofacial function and malocclusion were carried out at the same time and for this reason we do not know which characteristic precedes what (or, indeed, which characteristic *causes* what).

## 7 CLINICAL IMPLICATIONS

Orofacial motor function is an understudied and underassessed area in speech‐language pathology. However, the high co‐occurrence of orofacial dysfunction and malocclusion among our participants illustrates the need to have a multiprofessional approach when working with children with persistent SSD and possibly more regular dental care, monitoring of occlusal development, and the ability for oral self‐clearance. Clinicians working with children with SSD need to be aware of co‐occurring orofacial dysfunctions that may influence important daily life functions such as chewing, nose breathing and swallowing saliva, and possibly speech interventions (Wren et al., 2016). Furthermore, chewing difficulties may influence gastrointestinal function and result in selective eating (N'Gom & Woda, 2002), if not discovered and communicated to caregivers. The co‐occurrence of orofacial dysfunction also highlights that professionals, working with children with SSD, need access to validated structured assessment tools that can offer an overall picture of orofacial function. The present extensive assessment procedure illustrates the complexity. If suspecting orofacial dysfunctions NOT‐S can be used as a screening, possibly in combination with bite blocks to assess jaw stability. These assessments form a comprehensive and easily accessible set of methods for clinical practice.

## 8 CONCLUSION

Children with SSD may be at a greater risk of poor orofacial functioning and malocclusion than same‐age peers. Among children with SSD, poorer orofacial functioning was related to an increased risk of also having a malocclusion. These results indicate the importance of assessing and treating children with SSD using a multiprofessional approach.

## AUTHOR CONTRIBUTIONS

Åsa Mogren collected the data, drafted the manuscript, conceptualized the study, and defined the methodology. Anders Sand participated in analyzing the data, defining the methodology, and manuscript editing. Christina Havner collected the data and participated in conceptualizing the study and in manuscript editing. Lotta Sjögreen participated in conceptualizing the study, defining the methodology, and manuscript editing. Anna Westerlund participated in defining the methodology and in manuscript editing. Monica B. Agholme participated in conceptualizing the study and in manuscript editing. Anita McAllister conceptualized the study and participated in defining the methodology and in manuscript editing.

## CONFLICT OF INTEREST

The authors declare no conflict of interest.

## ETHICS STATEMENT

This study was approved by the Regional Ethical Review Board in Gothenburg (Dnr. 363‐14). All participants received both oral and written information about the study. The children received a simplified version of the information, including pictorial support. Adolescents were involved in a discussion about their participation. The parents signed an informed consent form allowing the research and publication of the results before any assessments were initiated.

## Supporting information

Supporting information.

Supporting information.

## Data Availability

The authors confirm that the data supporting the findings of this study are available within the article. The data will be shared on reasonable request to the corresponding author.
