# Supplementary material for: Children and adolescents with speech sound disorders are more likely to have orofacial dysfunction and malocclusion
Source: Clin Exp Dent Res. 2022 Jun 20;8(5):1130–41. doi: 10.1002/cre2.602 (PMC9562821; doi:10.1002/cre2.602)
Supplement: Supplementary file 1 — Supporting information. [file CRE2-8-1130-s001.docx]

*Supplementary table 1.* Feature list used in differential diagnostics for the different motor speech disorders. Data on voice deviation and percentage consonants correct (PCC) is added. Calculation of percentages was not used for Articulation Impairment (AI) and Developmental Dysarthria (DD) as the subgroups contained few participants.

|  | **All N =61** | **CAS N=25** | **SMD N=23** | **sCAS N=9** | **AI N=3** | **DD N=1** |
| --- | --- | --- | --- | --- | --- | --- |
| Mean age (min-max) | 8:5  (6:0-16:7) | 7:8 (6:0-15:3) | 8:8 (6:1-16:1) | 9:2 (6:0-16:7) | 10:7 (9:2-13:5) | 7:3 |
| Consonant distortion n (%) | 60 (98%) | 25 (100%) | 22 (96%) | 9 (100%) | 3 | 1 |
| Vowel error n (%) | 51 (84%) | 25 (100%) | 17 (74%) | 8 (89%) | 0 | 1 |
| Stress error n (%) | 31 (51%) | 23 (92%) | 6 (26%) | 2 (22%) | 0 | 0 |
| Syllable segregation n (%) | 17 (28%) | 12 (48%) | 2 (9%) | 2 (22%) | 0 | 1 |
| Groping n (%) | 23 (38%) | 17 (68%) | 3 (13%) | 3 (33%) | 0 | 0 |
| Intrusive schwa n (%) | 15 (25%) | 9 (36%) | 5 (22%) | 1 (9%) | 0 | 0 |
| Voicing error n (%) | 36 (59%) | 21 (84%) | 9 (39%) | 5 (55%) | 1 | 0 |
| Slow rate n (%) | 5 (8%) | 4 (16%) | 0 | 0 | 0 | 1 |
| Increased difficulties with multisyllabic words n (%) | 1 (2%) | 0 | 1 (4%) | 0 | 0 | 0 |
| Nasal resonance n (%) | 31 (51%) | 14 (56%) | 12 (52%) | 3 (33%) | 1 | 1 |
| Difficulties achieving initial articulatory configurations n (%) | 31 (51%) | 19 (76%) | 4 (17%) | 7 (78%) | 0 | 1 |
| Numbers of features Mean ± (min-max) | 4.9 ±2.0 (1-9) | 6.8 ±1.2 (5-9) | 3.5 ±0.9 (2-6) | 4.4 ±1.0 (3-6) | 1.7 ±1.1 (1-3) | 6 |
| Inconsistency n (%) | 37 (61%) | 25 (100%) | 6 (26%) | 7 (78%) | 0 | 0 |
| **Other** |  |  |  |  |  |  |
| Voice deviation (hoarsness, pitch, weak voice) n (%) | 24 (39%) | 9 (36%) | 9 (39%) | 4 (44%) | 2 | 0 |
| PCC mean % | 66 | 52.9 | 78.3 | 66.9 | 88.7 | 57 |

*Note.* Childhood Apraxia of Speech (CAS) was confirmed if the child had five features from the list + inconsistency according to Iuzzini-Seigel (2017), Speech Motor Delay (SMD), Articulation Impairment (AI) and Developmental Dysarthria (DD) was used as described in Shriberg et al (2010; 2019), Suspected Childhood Apraxia of Speech (sCAS) was used if the child did not fulfil all the proposed criteria of CAS but still had a motor speech disorder that included several CAS features.

Iuzzini-Seigel, J., Murray, E. (2017). Speech Assessment in Children With Childhood Apraxia of Speech. *Perspectives of the ASHA Special Interest Groups, 2*, 47-60. doi:10.1044/persp2.SIG2.47

Shriberg, L. D., Campbell, T. F., Mabie, H. L., & McGlothlin, J. H. (2019). Initial studies of the phenotype and persistence of speech motor delay (SMD). *Clinical Linguistics & Phonetics, 33*(8), 737-756. doi:10.1080/02699206.2019.1595733

Shriberg, L. D., Fourakis, M., Hall, S. D., Karlsson, H. B., Lohmeier, H. L., McSweeny, J. L., . . . Wilson, D. L. (2010). Extensions to the Speech Disorders Classification System (SDCS). *Clinical Linguistics & Phonetics, 24*(10), 795-824. doi:10.3109/02699206.2010.503006
