# Supplementary material for: Children and adolescents with speech sound disorders are more likely to have orofacial dysfunction and malocclusion
Source: Clin Exp Dent Res. 2022 Jun 20;8(5):1130–41. doi: 10.1002/cre2.602 (PMC9562821; doi:10.1002/cre2.602)
Supplement: Supplementary file 2 — Supporting information. [file CRE2-8-1130-s002.docx]

*Supplementary table 2*. Orofacial function and malocclusion in females and males with typical speech development (TSD) and in younger and older children with TSD.

| **Variable** | **TSD**  **Females**  **(*n* = 19)** | **TSD**  **Males**  **(*n* = 25)** | **TSD**  **Age 6:00-7:11**  **(*n*=13)** | **TSD**  **Age 8:00-12:2**  **(n=31)** | **TSD**  **All**  **(*n* =44)** |
| --- | --- | --- | --- | --- | --- |
| Age, year:month, mean ± SD | 8:6 ± 1:6 | 8:9 ± 1:5 | 6:9 ± 0:6 | 9:6 ± 1:0 | 8:7 ± 1:6 |
| NOT-S^I^ Total Score (0-12), mean ± SD, | 0.16 ± 0.37 | 0.32 ± 0.56 | 0.23 ± 0.44 | 0.26 ± 0.51 | 0.25 ± 0.49 |
| Maximum bite force, Newton, mean ± SD | 294 ±76 | 387 ± 126 | 291 ± 62 | 370 ± 126 | 346 ± 116 |
| Jaw stability^I^, bite block level (0-6) mean ± SD | 5.6 ± 0.79 | 5.14 ± 0.91 | 5.3 ± 0.75 | 5.3 ± 0.93 | 5.3 ± 0.87 |
| Chewing efficiency^II^, SDHue, mean ± SD, | 0.16 ± 0.07 | 0.13 ± 0.04 | 0.17 ± 0.06 | 0.13 ± 0.06 | 0.14 ± 0.06 |
| Sensory-motor function^I^, (0-8), mean ± SD | 7.11 ± 96 | 7.00 ± 0.95 | 7.15 ± 0.80 | 7.00 ± 1.02 | 7.05 ± 0.94 |
| No malocclusion *n* (%) | 15 (79) | 16 (64) | 11 (85) | 20 (64) | 32 (73) |
| Malocclusion^IV^ *n* (%) | 4 (21) | 9 (36) | 2 (15) | 11(36) | 13 (29) |

*Note.* ^I^ Ordinal scale data, for more detailed distribution of data, see Figure1. ^II^ One missing value in the male group and in age group 8:00-12:2.
